# Higher Risk of Wheeze in Female than Male Smokers. Results from the Swedish GA^2^LEN Study

**DOI:** 10.1371/journal.pone.0054137

**Published:** 2013-01-24

**Authors:** Anders Bjerg, Linda Ekerljung, Jonas Eriksson, Inga Sif Ólafsdóttir, Roelinde Middelveld, Karl A. Franklin, Bertil Forsberg, Kjell Larsson, Jan Lötvall, Kjell Torén, Sven-Erik Dahlén, Bo Lundbäck, Christer Janson

**Affiliations:** 1 Krefting Research Centre, Department of Internal Medicine, University of Gothenburg, Göteborg, Sweden; 2 The OLIN Studies, Department of Medicine, Sunderby Central Hospital of Norrbotten, Luleå, Sweden; 3 Occupational and Environmental Medicine, Sahlgrenska School of Public Health, University of Gothenburg, Göteborg, Sweden; 4 Centre for Allergy Research and Institute of Environmental Medicine, Karolinska Institutet, Stockholm, Sweden; 5 Department of Surgery, Umeå University, Umeå, Sweden; 6 Environmental and Occupational Medicine, Department of Public Health and Clinical Medicine, Umeå University, Umeå, Sweden; 7 Lung and Allergy Research, Institute of Environmental Medicine, Karolinska Institutet, Stockholm, Sweden; 8 Department of Respiratory Medicine and Allergology, Sahlgrenska University Hospital, Göteborg, Sweden; 9 Department of Medical Sciences: Respiratory Medicine and Allergology, Uppsala University, Uppsala, Sweden; Universität Bochum, Germany

## Abstract

**Background:**

Women who smoke have higher risk of lung function impairment, COPD and lung cancer than smoking men. An influence of sex hormones has been demonstrated, but the mechanisms are unclear and the associations often subject to confounding. This was a study of wheeze in relation to smoking and sex with adjustment for important confounders.

**Methods:**

In 2008 the Global Allergy and Asthma European Network (GA^2^LEN) questionnaire was mailed to 45.000 Swedes (age 16–75 years), and 26.851 (60%) participated. “Any wheeze”: any wheeze during the last 12 months. “Asthmatic wheeze”: wheeze with breathlessness apart from colds.

**Results:**

Any wheeze and asthmatic wheeze was reported by 17.3% and 7.1% of women, vs. 15.8% and 6.1% of men (both p<0.001). Although smoking prevalence was similar in both sexes, men had greater cumulative exposure, 16.2 pack-years vs. 12.8 in women (p<0.001). Most other exposures and characteristics associated with wheeze were significantly overrepresented in men. Adjusted for these potential confounders and pack-years, current smoking was a stronger risk factor for any wheeze in women aged <53 years, adjusted odds ratio (aOR) 1.85 (1.56–2.19) vs. 1.60 (1.30–1.96) in men. Cumulative smoke exposure and current smoking each interacted significantly with female sex, aOR 1.02 per pack-year (p<0.01) and aOR 1.28 (p = 0.04) respectively. Female compared to male current smokers also had greater risk of asthmatic wheeze, aOR 1.53 vs. 1.03, interaction aOR 1.52 (p = 0.02). These interactions were not seen in age ≥53 years.

**Discussion:**

In addition to the increased risk of COPD and lung cancer female, compared to male, smokers are at greater risk of significant wheezing symptoms in younger age. This became clearer after adjustment for important confounders including cumulative smoke exposure. Estrogen has previously been shown to increase the bioactivation of several compounds in tobacco smoke, which may enhance smoke-induced airway inflammation in fertile women.

## Introduction

The detrimental effects of tobacco smoking on respiratory health are well known. Most importantly, smoking significantly accelerates lung function decline, and increases the risk of lung cancer and chronic obstructive pulmonary disease (COPD) [1]. Lung function is more negatively affected by smoking in women compared to men. Women who smoke are at higher risk of attenuated lung growth in adolescence [2], [3], airway narrowing and increased bronchial hyperresponsiveness [4], [5], and later hospitalizations for COPD than male smokers [6].

Lung function, as measured by a single spirometry, however does not necessarily correlate well with respiratory symptoms in the individual [7]. In asthma, the temporal variability of airway calibre and symptoms may not be detected upon a single lung function measurement. In COPD despite fixed airway obstruction being the hallmark of disease symptoms, general health status and exercise capacity all correlate poorly with lung function impairment [7], [8]. Respiratory symptoms can thus be a more sensitive and relevant indicator of illness, and may precede subsequent lung function decline [4].

Some recent findings suggest that hormonal factors play an important role in the increased susceptibility to COPD and lung cancer in women [9], [10]. However, it is also well known that smoking behaviour, e.g. age at smoking initiation and pack-years, and other exposures from occupation and general lifestyle differ between men and women [6], [11]. Also basic characteristics (e.g. age, weight, BMI and co-morbidities) may significantly confound the associations between sex, smoking and respiratory disease [12]. Such confounding is a likely explanation for the early controversies as to which sex is more susceptible to smoking [13] and have not been accounted for in previous studies of respiratory symptoms [3], [14]. Thus, whether women who smoke truly are at greater risk of wheeze than male smokers remains unclear.

In several high-income countries smoking prevalence is increasing in women, and concurrently, smoking-related diseases such as COPD are projected to have a female predominance within ten years [15]. If, in addition, women are more susceptible to respiratory illness from smoking, these trends could be accelerated.

In a previous study of a subsample within the Swedish Global Allergy and Asthma European Network (GA^2^LEN) survey, there were signs of a greater impact of smoking on wheeze in women than in men [16]. Using the full Swedish GA^2^LEN database, the present aim was to study sex-specific effects of smoking on wheezing in adults, with adjustment for relevant confounders.

## Materials and Methods

### Ethics statement

Ethical approval was granted by the Regional Ethical Committee at Uppsala University, Uppsala, Sweden.

### Study population

The study population of the Swedish GA^2^LEN questionnaire study has been described in detail previously [16]. In brief, a random sample of 45.000 adults aged 16–75 years living in four areas of Sweden (Göteborg, Stockholm, Umeå and Uppsala) were invited to a postal questionnaire survey in 2008, and 26.851 (60%, 53% female) complete questionnaires were included in the analysis.

### Questionnaire and definitions

The questionnaire included items on respiratory symptoms, anthropometric data, education level and employment, and environmental exposures including smoking. The core symptom questions were the European Community Respiratory Health Survey (ECRHS) items, which have been thoroughly evaluated clinically, including lung function measurements and bronchial hyperresponsiveness [17], [18]. The questions have been translated and back-translated, and have been used repeatedly in previous Swedish studies [19], [20].

The majority of definitions have been published previously [16], and only those of special relevance to the present paper are given below.

#### Sex/gender

As this was a study of biological differences between the sexes in the impact of smoking, the term sex is used thoroughly. The term gender is more relevant in social science.

#### Any wheeze

“Have you had wheezing or whistling in your chest at any time in the last 12 months?”.

#### Asthmatic wheeze

Affirmative answers to *any wheeze*, and “Have you been at all breathless when the wheezing noise was present?” and “Have you had this wheezing or whistling when you did not have a cold?”.

#### Chronic rhinosinusitis (CRS)

Following the EP3OS criteria [21], the presence of at least two of: (i) nasal blockage, (ii) nasal discharge, (iii) facial pain or pressure or (iv) reduction in sense of smell with at least one of the symptoms being nasal blockage or nasal discharge.

*Menopause*: The mean age at menopause was extrapolated to 53 years, using previous data from Sweden [22].

#### Ever smoking

“Have you ever smoked one or more cigarettes per day for more than one year?”.

#### Current smoking

Affirmative answer to *ever smoking*, and “…If so, have you at all smoked during the last month?”.

#### Pack-years

Number of cigarettes smoked per day divided by 20, times number of years' smoking.

#### Outdoor air irritating

“How often do you find the air in your residential area irritating?”.

#### Vehicle exhaust annoying

“How troublesome are the traffic exhausts in your residential area?”.

#### Traffic exposure

“How many minutes each weekday are you surrounded by city traffic?”.

#### Damp home

Signs of indoor moisture damage or visible moulds.

**Vapors, gas, dust or fumes** “Have you ever had a working place with much vapors, gas, dust or fume in the air?”.

### Statistical analyses

Comparisons of prevalence were performed using the two-sided chi^2^-test. For the continuous variables the Mann-Whitney U-test was used for comparisons of means due to lack of normal distribution. P-values<0.05 were considered statistically significant. Risks were expressed as univariate prevalence ratios (PR). Logistic regression was used to calculate multivariate adjusted odds ratios (OR) and generalized linear modelling was used to calculated adjusted PR with 95% confidence intervals.

The multivariate analyses in ages 16–52 and ages 53–75 respectively were performed separately for the outcomes any wheeze and asthmatic wheeze. Statistical interactions with female sex were tested for three smoking indices, each presented in a separate column: I) ever smoking (adjusted for pack-years); II) current smoking (adjusted for pack-years) and III) pack-years, resulting in three models (Ia-IIIa) for any wheeze and three models (Ib-IIIb) for asthmatic wheeze. As an example, model Ia thus included the variables female sex, ever smoking and number of pack-years, and the interaction term female sex*ever smoking. All baseline characteristics and exposures (table 1) were added to the multivariate models, and statistically non-significant (p>0.2) independent variables were then removed stepwise from the models.

**Table 1:** Basic characteristics of the study population.

	All	Females	Males	P
	(n = 27861)	(n = 14678)	(n = 12173)	
Airway symptoms				
Any wheeze	16.6	17.3	15.8	0.001
Asthmatic wheeze	6.7	7.1	6.1	0.001
Nocturnal symptoms	31.3	35.8	25.8	<0.001
Ever asthma	12.6	13.7	11.4	<0.001
Current asthma	7.1	7.9	6.1	<0.001
Chronic bronchitis	12.0	11.7	12.2	0.237
Characteristics				
Age	43.8 (16.1)	43.2 (16.2)	44.6 (16.0)	<0.001
BMI	24.7 (4.14)	24.1 (4.27)	25.5 (3.84)	<0.001
Chronic rhinosinusitis	8.5	8.7	8.3	0.201
Secondary schooling	86.6	87.0	86.1	0.047
University education	49.9	52.5	46.9	<0.001
Vapors, gas, dust or fumes	36.7	25.1	50.7	<0.001
Smoking				
Ever	39.4	39.4	39.2	0.831
Pack-years	14.3 (14.5)	12.8 (12.7)	16.2 (16.3)	<0.001
Age at initiation	17.1 (4.26)	17.1 (4.21)	17.1 (4.32)	0.423
Current	13.9	14.5	13.3	0.004
Pack-years	15.8 (15.9)	14.3 (13.5)	18.0 (18.4)	<0.001
Age at initiation	17.1 (4.62)	17.0 (4.38)	17.1 (4.9)	0.276
Ex	25.4	24.9	26.0	0.039
Pack-years	13.3 (13.5)	11.6 (11.8)	15.2 (14.9)	<0.001
Age at initiation	17.0 (3.90)	17.1 (4.04)	16.9 (3.74)	0.770

Prevalence (%) and means (SD), P-value for males vs. females (chi^2^-test or Mann-Whitney U-test).

**Figure 1:**
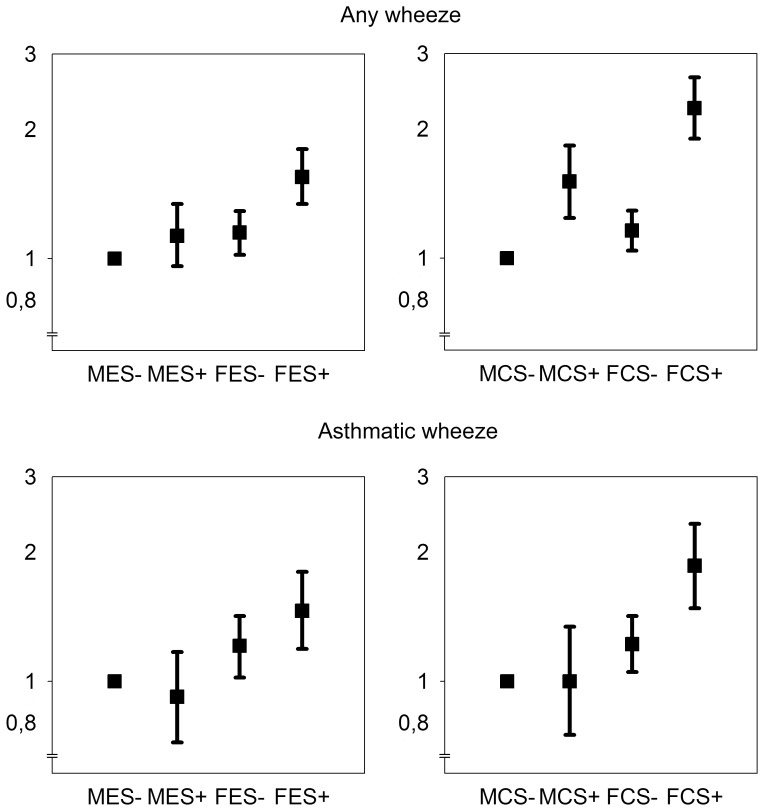
Adjusted risks of wheeze from sex and smoking in ages 16–52 years by categorical analysis. Each graph displays the adjusted odds ratios and 95% confidence intervals for wheeze from four mutually exclusive categories based on sex and smoking status. M: male; F: female; ES: ever smokers; CS: current smokers; + “yes”; − “no”. Multivariate models were obtained from table 3, and the categorical variables were substituted for the interaction terms. Note the logarithmic Y axes, and the breaks between Y = 0 to Y = 0,7.

For figure 1, one variable with four mutually exclusive categories of sex (male/female) and smoking status (yes/no) were substituted for the interaction terms, using the same multivariate models for ever smoking (models Ia and IIa) and current smoking (models Ib and IIb). Analyses were performed using PASW Statistics 18.0 (IBM Corp, New York, USA and Stata 12.0, Stata Corp, College Station, Texas, USA).

## Results

The prevalence of respiratory symptoms common in asthma was higher in women compared to men, whereas chronic bronchitis was equally common in both sexes (table 1). Any wheeze, asthmatic wheeze and current asthma were reported by 17.3%, 7.1% and 7.9% of women, compared to 15.8%, 6.1% and 6.1% of men (all p<0.001).

There were considerable differences by sex in the prevalence of potential risk factors for wheeze (table 1). Mean age and BMI were higher in men, and fewer had a university education (all p<0.001). Exposure to vapors, gas, dust and fumes was twice as common in men. The prevalence of chronic rhinosinusitis was similar in women and men, 8.7% and 8.3% respectively (p = 0.20).

Ever having smoked was reported by 39.4%, similar in both sexes. Current smoking was slightly more common in women (14.5% vs. 13.3%, p<0.01). Whereas age at smoking initiation was very similar in the sexes, men had higher cumulative exposure to tobacco smoke, p<0.001. As expected, subjects reporting any wheeze reported significantly higher prevalence of other respiratory symptoms (table 2). Subjects with any wheeze also had slightly higher mean BMI, lower level of education, and were more likely to report exposure to vapors, gas, dust or fumes and smoking.

**Table 2:** Characterisation of subjects with and without any wheeze.

	Any wheeze	No wheeze	
	(n = 4362)	(n = 21908)	P
Airway symptoms			
Asthmatic wheeze	40.1	-*	<0.001
Nocturnal symptoms	65.4	24.3	<0.001
Ever asthma	42.8	6.7	<0.001
Current asthma	32.2	2.1	<0.001
Chronic bronchitis	33.8	7.4	<0.001
Characteristics			
Age	44.1 (15.9)	43.0 (15.8)	<0.001
BMI	25.9 (4.77)	24.5 (4.05)	<0.001
Chronic rhinosinusitis	21.4	5.8	<0.001
Secondary schooling	85.0	87.2	<0.001
University education	46.3	51.1	<0.001
Vapors, gas, dust or fumes	48.4	34.3	<0.001
Smoking			
Ever	51.5	36.9	<0.001
Pack-years	17.7 (16.4)	13.2 (13.8)	<0.001
Age at initiation	17.1 (4.18)	16.7 (4.34)	<0.001
Current	25.0	11.6	<0.001
Pack-years	20.2 (17.5)	14.0 (14.8)	<0.001
Age at initiation	16.5 (4.32)	17.3 (4.72)	<0.001
Ex	25.9	25.1	0.266
Pack-years	14.9 (14.6)	12.8 (13.1)	<0.001
Age at initiation	17.0 (4.35)	17.0 (3.85)	<0.001

Prevalence (%) and means (SD), P-value for any wheeze vs. no wheeze (chi^2^-test or Mann-Whitney U-test).

* Any wheeze was included in the definition of asthmatic wheeze.

The unadjusted risks of any wheeze and asthmatic wheeze from ever and current smoking were similar in men and women (table 3). A greater risk of asthmatic wheeze from current smoking was seen in women (interaction by sex p = 0.04). The increased risk in women compared to men was more pronounced in the ages 16–52, PR 1.90 (95% CI 1.61–2.25) vs. PR 1.37 (1.08–1.75) (p = 0.03). In older subjects this interaction was not seen.

**Table 3:** Risk of wheeze from ever and current smoking, stratified by sex and age.

		Any wheeze	Asthmatic wheeze
		RR (95% CI)	RR (95% CI)
Ever smoking			
	Males	1.64 (1.50–1.78)	1.48 (1.29–1.71)
All	Females	1.60 (1.49–1.72)	1.59 (1.41–1.79)
	P	0.811	0.438
	Males	1.58 (1.41–1.76)	1.41 (1.17–1.70)
Age 16–52	Females	1.56 (1.43–1.71)	1.51 (1.33–1.83)
	P	0.971	0.542
	Males	1.94 (1.65–2.27)	1.87 (1.43–2.46)
Age 53–75	Females	1.65 (1.45–1.89)	1.89 (1.50–2.38)
	P	0.180	0.933
Current smoking			
	Males	1.99 (1.81–2.18)	1.55 (1.30–1.85)
All	Females	2.12 (1.97–2.29)	1.94 (1.69–2.22)
	P	0.179	0.044
	Males	1.81 (1.59–2.06)	1.37 (1.08–1.75)
Age 16–52	Females	2.04 (1.85–2.25)	1.90 (1.61–2.25)
	P	0.112	0.026
	Males	2.29 (1.99–2.63)	1.87 (1.43–2.46)
Age 53–75	Females	2.28 (2.01–2.58)	2.04 (1.62–2.56)
	P	0.831	0.616

Risks displayed as risk ratios with 95% confidence intervals. P-values for difference by sex (current smoking*sex interaction term).

Multivariate models were obtained by entering all the surveyed potential risk factors for any and asthmatic wheeze, respectively, and then stepwise removing risk factors with p>0.2 for the studied outcome. Secondary schooling was kept in the model to control for level of education. The age groups 16–52 and 53–75 years were analysed separately.

In concordance with the unadjusted analyses, the adjusted odds ratios for any wheeze from current smoking was higher in women than in men aged 16–52, OR 2.00 (1.66–2.40) vs. 1.70 (1.36–2.13), adjusted for secondary schooling; irritation from air; vapor, gas, dust or fume exposure and pack-years. Similarly, the odds ratio per ten pack-years of smoking was higher in women, 1.36 (1.27–1.46) than in men, 1.21 (1.12–1.31). For asthmatic wheeze, the difference by sex in the effect of current smoking was even larger, OR 1.53 (1.21–1.92) in women vs. OR 1.03 (0.75–1.40) in men. These differences were not seen in subjects aged 53–75 years. The adjusted PR for the association between any wheeze and current smoking was 1.71 (1.50–1.96) vs. 1.54 (1.31–1.83) in women and men, respectively.

To test the statistical significance of the differences by sex, interaction terms for sex*smoking (ever smoking; current smoking; or pack-years, respectively) were included in multivariate models. Separate analyses were performed for the age groups 16–52 (table 4) and 53–75 (table 5) years.

**Table 4:** Adjusted risks of wheeze by multivariate analysis in subjects aged 16–52 years.

Risk factor*	Any wheeze**			Asthmatic wheeze***		
	Model 1	Model 2	Model 3	Model 4	Model 5	Model 6
	OR (95% CI)	OR (95% CI)	OR (95% CI)	OR (95% CI)	OR (95% CI)	OR (95% CI)
Female sex	0.97 (0.73–1.27)	0.91 (0.68–1.22)	1.16 (1.05–1.29)	0.91 (0.62–1.35)	0.81 (0.53–1.23)	1.28 (1.11–1.49)
BMI (vs. 20–24.9)						
<20	0.88 (0.75–1.03)	0.86 (0.73–1.01)	0.88 (0.75–1.03)	0.84 (0.66–1.06)	0.83 (0.66–1.05)	0.84 (0.66–1.06)
25–30	1.24 (1.11–1.38)	1.26 (1.13–1.40)	1.25 (1.12–1.39)	1.20 (1.03–1.39)	1.20 (1.03–1.40)	1.20 (1.03–1.40)
>30	2.04 (1.76–2.37)	2.09 (1.80–2.42)	2.04 (1.76–2.37)	1.76 (1.44–2.15)	1.77 (1.45–2.17)	1.76 (1.44–2.15)
Secondary schooling	1.12 (0.94–1.34)	1.16 (0.97–1.39)	1.13 (0.95–1.35)	1.00 (0.79–1.27)	1.02 (0.81–1.29)	1.01 (0.80–1.28)
Chronic rhinosinusitis	3.25 (2.87–3.68)	3.20 (2.82–3.63)	3.29 (2.90–3.72)	2.84 (2.42–3.33)	2.80 (2.38–3.29)	2.85 (2.42–3.34)
Smoking						
Ever	0.95 (0.68–1.33)	-	-	0.69 (0.43–1.13)	-	-
Current	-	1.18 (0.79–1.76)	-	-	0.66 (0.36–1.20)	-
Per pack-year	1.02 (1.01–1.03)	1.02 (1.01–1.02)	1.00 (0.99–1.02)	1.02 (1.01–1.03)	1.01 (1.01–1.02)	1.00 (0.98–1.03)
Smoking*female sex interaction term						
Ever	1.19 (0.98–1.44)	-	-	1.32 (1.00–1.75)	-	-
Current	-	1.28 (1.01–1.62)	-	-	1.52 (1.08–2.13)	-
Per pack-year	-	-	1.02 (1.01–1.03)	-	-	1.01 (0.99–1.03)

Multivariate odds ratios (OR) with 95% confidence intervals (CI) were calculated for any wheeze (models 1–3) and asthmatic wheeze (models 4–6). Interaction with sex was tested separately for each category of smoking: ever smoking (adjusted for pack-years) in models 1 and 4; current smoking (adjusted for pack-years) in models 2 and 5; and number of pack-years in models 3 and 6.

* All factors significantly associated with the respective wheeze outcomes in univariate analysis were entered into the model, and then removed stepwise if p>0.2, yielding the final models presented below.

** Models 1–3 included female sex, BMI (<20; 25–30; >30; vs. 20–24.9); secondary schooling; perceived irritation from outdoor air (sometimes; daily; vs never); minutes of daily traffic exposure (30–60; >60; vs. <30); damp in the home; work exposure to vapors, gas, dust and fumes; chronic rhinosinusitis, and smoking as listed.

*** Models 4–6 included female sex, BMI (<20; 25–30; >30; vs. 20–24.9); secondary schooling; perceived irritation from outdoor air (sometimes; daily; vs never); work exposure to vapors, gas, dust and fumes; chronic rhinosinusitis, and smoking as listed.

**Table 5:** Adjusted risks of wheeze by multivariate analysis in subjects aged 16–52 years.

Risk factor*	Any wheeze**			Asthmatic wheeze***		
	Model 1	Model 2	Model 3	Model 4	Model 5	Model 6
	OR (95% CI)	OR (95% CI)	OR (95% CI)	OR (95% CI)	OR (95% CI)	OR (95% CI)
Female sex	1.33 (0.82–2.15)	1.59 (1.05–2.41)	1.35 (1.12–1.61)	0.99 (0.49–2.00)	1.67 (0.92–3.02)	1.38 (1.06–1.80)
BMI (vs. 20–24.9)						
<20	0.74 (0.49–1.11)	0.65 (0.43–0.99)	0.72 (0.48–1.09)	1.09 (0.65–1.83)	1.04 (0.62–1.75)	1.09 (0.65–1.82)
25–30	1.63 (1.40–1.90)	1.70 (1.45–1.99)	1.63 (1.39–1.90)	1.30 (1.04–1.63)	1.32 (1.05–1.65)	1.29 (1.03–1.62)
>30	2.62 (2.16–3.18)	2.87 (2.36–3.49)	2.59 (2.13–3.14)	2.07 (1.59–2.70)	2.13 (1.63–2.78)	2.04 (1.56–2.66)
Secondary schooling	1.10 (0.94–1.29)	1.17 (0.99–1.37)	1.11 (0.95–1.31)	0.89 (0.72–1.10)	0.92 (0.74–1.14)	0.90 (0.73–1.11)
Chronic rhinosinusitis	4.75 (3.86–5.86)	4.60 (3.72–5.68)	4.73 (3.84–5.83)	4.31 (3.38–5.49)	4.22 (3.31–5.38)	4.31 (3.38–5.49)
Smoking						
Ever	1.21 (0.75–1.95)	-	-	0.81 (0.40–1.64)	-	-
Current	-	2.58 (1.55–4.29)	-	-	1.50 (0.72–3.13)	-
Per pack-year	1.02 (1.01–1.02)	1.02 (1.01–1.20)	1.01 (0.997–1.02)	1.02 (1.01–1.02)	1.02 (1.01–1.02)	1.00 (0.99–1.02)
Smoking*female sex interaction term						
Ever	1.10 (0.83–1.46)	-	-	1.36 (0.90–2.05)	-	-
Current	-	0.96 (0.70–1.31)	-	-	0.98 (0.63–1.52)	-
Per pack-year	-	-	1.01 (1.00–1.02)	-	-	1.01 (1.00–1.02)

Multivariate odds ratios (OR) with 95% confidence intervals (CI) were calculated for any wheeze (models 1–3) and asthmatic wheeze (models 4–6). Interaction with sex was tested separately for each category of smoking: ever smoking (adjusted for pack-years) in models 1 and 4; current smoking (adjusted for pack-years) in models 2 and 5; and number of pack-years in models 3 and 6.

* All factors significantly associated with the respective wheeze outcomes in univariate analysis were entered into the model, and then removed stepwise if p>0.2, yielding the final models presented below.

** Models 1–3 included female sex, BMI (<20; 25–30; >30; vs. 20–24.9); secondary schooling; perceived irritation from outdoor air (sometimes; daily; vs never); minutes of daily traffic exposure (30–60; >60; vs. <30); damp in the home; work exposure to vapors, gas, dust and fumes; chronic rhinosinusitis, and smoking as listed.

*** Models 4–6 included female sex, BMI (<20; 25–30; >30; vs. 20–24.9); secondary schooling; perceived irritation from outdoor air (sometimes; daily; vs never); work exposure to vapors, gas, dust and fumes; chronic rhinosinusitis, and smoking as listed.

In subjects aged 16–52 the multivariate analyses revealed clear statistical interactions between female sex and smoking on the risk of wheeze (table 4). Adjusted for other risk factors and pack years female current smokers had a greater risk of any wheeze than male current smokers, OR 1.28 (1.01–1.62), interaction p = 0.04. Per pack-year the risk of any wheeze increased in female compared to male smokers by OR 1.02 (1.01–1.03), interaction p<0.01. Similarly, adjusted for other risk factors and pack-years, female compared to male ever and current smokers were at greater risk of asthmatic wheeze, OR 1.32 (1.00–1.75) p = 0.047 and OR 1.52 (1.08–2.13) p = 0.02. The inclusion of cumulative smoke exposure (pack-years) in the model strengthened the female sex*smoking interactions, but dissipated the effects of ever/current smoking alone. Grouping pack-years into 10 or 20-year categories yielded similar, increasing dose-response results, but few subjects in the higher exposure groups limited the analysis.

The majority of tested risk factors other than smoking were statistically significantly associated with wheeze and asthmatic wheeze. Chronic rhinosinusitis was a major determinant of wheeze, OR 3.2–3.3, and asthmatic wheeze, OR 2.8–2.9, and increasing BMI was linearly associated to both outcomes. The interactions of female sex with ever and current smoking observed in the younger age group was not seen in subjects aged 53–75 (table 5).

A categorical variable was substituted for the dichotomous variables on sex and smoking, using the same multivariate models. The results for subjects aged 16–52 are presented in figure 1. Compared to male non-smokers, female non-smokers had a slightly but significantly increased risk of wheeze and asthmatic wheeze. In men, current smoking significantly increased the risk of wheeze but not asthmatic wheeze. The joint effect of female sex and ever/current smoking was clearly greater than that expected on a multiplicative scale, confirming the interactions observed in table 3.

The interaction of female sex with smoking was similar in subjects initiating smoking before and after 20 years of age. The interaction was further explored in several different age and BMI groups with no findings of further interactions by these variables. Adding body weight or height to the multivariate models did not change the observed interactions of smoking by sex. The interaction with smoking disappeared when weight (adjusted for height and sex) or height (adjusted for weight and sex) was substituted for sex.

## Discussion

In this large, population-based study female smokers were at greater risk of wheeze compared to male smokers. For every ten pack-years smoked, the risk of any wheeze was 14% greater in women. This was clear after adjustment for total smoke exposure and several other important risk factors for wheeze. This interaction of female sex and smoking was most consistent in subjects younger than 53 years, and was not modified by height, weight, BMI, or age at smoking initiation. Neither was it explained by lower body mass or smaller lung surface (estimated by height) in women.

Little is presently known about sex-specific effects of smoking on respiratory symptoms. One very large study in Norway found a positive interaction between female sex and smoking on episodic wheeze and breathlessness [14]. However, these findings were only adjusted for pack-years and age and as our and other studies show [3], [12], these associations are confounded by several important factors pertaining to smoking behaviour, comorbidity, anthropometric indices and environmental exposures which are not evenly distributed between the sexes. Our adjusting for these factors in fact helped to further unravel the interaction effects.

The negative effect of smoking on lung function is greater in female than in male smokers. Several studies have demonstrated increased risk of airway obstruction measured by spirometry [2], [4], [13] and increased risk of COPD and hospitalizations for COPD exacerbations [11]. Attenuated lung growth before the reaching of peak lung function at age 18–20 has been demonstrated in smoking female adolescents [2], [3]. The negative impact of smoking is however not limited to attenuation of lung growth since in our study the interaction of female sex with smoking was seen also in subjects initiating smoking after age 20. Rather, our findings underline the importance of total pack-years, which is often greater in early starters. Compared to a single spirometry measurement, questionnaire items have the advantage of being able to cover longer time spans, thus capturing occasional and variable symptoms. The downside to questionnaires is recall bias, but it seems unlikely that male smokers have worse recall of symptoms than female smokers and never-smoking peers of both sexes.

That the interaction was stronger in predominantly pre-menopausal ages [22] and was not explained by differences in height or weight between men and women point to biological differences by sex. To date, the mechanisms underlying an increased susceptibility in women to tobacco smoke have not been much studied. The bioactivity of cytochrome P450 (CYP) enzymes in the lungs is upregulated via estrogen in female compared to male smokers [10], causing more rapid metabolism e.g. of nicotine in females [23]. Several components of cigarette smoke are metabolised into more toxic substances by enzymes of the CYP family, so-called bioactivation [24]. It has been hypothesised that the increased CYP activity would thus cause greater accumulation of noxious metabolites in women, leading to more oxidative stress and tissue inflammation [9].

Increased inflammatory activity in turn is associated with bronchial hyperresponsiveness [25], which is enhanced in smoking women of fertile age, but not in men [5], [26]. Hyperresponsiveness, in turn, is firmly associated with wheezing symptoms [19], and is an important predictor of future decline in FEV1 and development of COPD [27], [28]. The observed interactions may thus be explained by increased bioactivation in women of certain tobacco smoke compounds, leading to airway inflammation, increased bronchial hyperresponsiveness and airway symptoms.

The strengths and limitations of the Swedish GA^2^LEN study have been discussed in detail previously [16]. Importantly, the study benefits from its large size and population-based design. A study of non-participants in a subsample confirmed the prevalence of symptoms, whereas smokers and men were slightly underrepresented among responders [29]. Thus, the prevalence of smoking may have been somewhat underestimated in the study, leading, if any, to a slight underestimation of the interactions observed among smokers in the study. As in all cross-sectional studies cause and effect cannot be determined from our results alone, and the terms “risk” and “odds” are used in their statistical context to define associations. The associations between smoking and wheeze however are well established throughout numerous studies. To mirror different aspects of respiratory morbidity the less specific measure wheeze, and a triad of symptoms, asthmatic wheeze [16], were chosen as the outcomes.

Only cigarette smoking was surveyed, but exclusive pipe and cigar smoking is highly uncommon in Sweden [30]. Recall may have led to underestimation of smoking especially in the older participants, however, this is unlikely to differ substantially between male and female ever smokers. The true age at menopause was not known so age 53 years, which was extrapolated from a previous large Swedish study of secular trends in menopause [22], was substituted. However, the interaction effect size was similar in ages <40 years and <44 years [16], but declined after age 50 years.

In conclusion, we found an interaction of female sex with smoking on the risk of wheeze. This increased susceptibility in women of predominantly fertile age points to hormonal factors. One plausible explanation is that bioactivation of certain tobacco smoke compounds into more noxious substances, which is enhanced by estrogen, may increase the risk of airway inflammation and wheezing symptoms. Whereas previous studies have shown an increased risk for COPD and lung cancer in smoking women compared to smoking men, our study shows that the risk of wheeze is increased already at lower ages.
